# The Abbey Service

**Published:** 1917-08-11

**Authors:** 


### The Abbey Service.

Those who were outside the Abbey immediately preceding the commencement of the Service of Intercession, especially if they approached it by St. James's Park, must have been cheered with a remembrance of peace-time and the good old days by seeing two open carriages, each with a pair of spanking horses, passing Wellington Barracks *en route* for the Abbey. As seen through the foliage they were in picturesque and pleasant contrast to the motor-cars which have superseded the carriage, and reminded the pedestrian that we have still nothing in this country for ceremonial occasions, which, for picturesque and other reasons, can equal a well-appointed carriage and horses. Those who arrived at the Abbey after 9.50 A.M. found the main entrances closed, for by this hour the transepts were crowded, the nave was filled, and seats were difficult to find anywhere, the congregation being very large. The reserved seats were so few as to be negligible, and the greater portion of them were occupied by wounded and injured men who came from the hospitals attended by their nurses. Many of the soldiers had lost one, and some two, legs, the legless men being carried on the back of a comrade. It was a very pathetic sight to see these poor fellows leaving the Abbey, though their wonderful cheerfulness and activity compelled the beholder to wish for the power to restore the missing limb or to supply whatever each warrior had lost through his devotion to the interests of his fellows and the good of the nation. The cumulative effect of a succession of legless men down the aisle, which might have been depressing otherwise, was heartening owing to the alertness and wonderful cheer of the poor fellows themselves.

Her Majesty the Queen was unfortunately absent through indisposition. The King, who was wearing naval uniform, was accompanied by Princess Mary, Princess Victoria, and Prince George. The Countess of Minto, Viscount Valentia, Lieut.-Colonel the Lord Stamfordham, and Commander Charles Cust, Bart., R.N., were in attendance. Forms of Prayer for Public Use on August 4 and 5, 1917, issued by authority, and published by the Society for Promoting Christian Knowledge, were placed on the seats in the Abbey. The form of service was Morning Prayer, and after the sermon by the Archbishop of Canterbury and a hymn, "O valiant hearts," Intercessions contained in the Forms of Prayer from page ix were recited, and the Blessing was pronounced by the Dean of Westminster, Bishop Ryle.

The music was, of course, good, but successive attendances at the Abbey on these occasions convince us that the choir at present is far too weak for so large and difficult a building for song. Indeed, it is time this was recognised, in justice to the national honour and the Empire, that the Choir may be strengthened without delay. At present, in the musical sense, the Abbey is altogether overshadowed by St. Paul's Cathedral, which is far and away better for the holding of great national services and commemorations. There were two hymns—Doddridge's hymn, "O God of Bethel," which commenced the service, and was quaint, attractive, and convincing, as it ever is; and Mr. John S. Arkwright's new war hymn, worthy of so all-absorbing an occasion, "O valiant hearts." This was sung to the tune of "Benediction," used for Hymn 31, "Saviour, again," in "Hymns Ancient and Modern." It is a beautiful hymn with beautiful words, and those of us who have given of our best to the country and lost dear ones in its cause were helped by the words and touched to the quick. Indeed, the impression made by this hymn was so forcible and impressive as to leave few dry eyes in the Abbey at its conclusion. It is a hymn which ought to be used regularly at the services in every hospital chapel throughout the Empire, and in all churches where the clergy are spiritually minded and in close touch with their people. The words of this hymn are given on page iv.

The special Psalms were the 24th and 46th. The Lessons were Isaiah xxxv. and Romans viii. 18. All the music in the service was English. The Te Deum was sung to Gray in G, and the Benedictus to Cobb in G. The Anthem after the third Collect, set to music by Dr. Walford Davies, was taken from the Sarum Primer 1558:

God be in my head, and in my understanding,
God be in mine eyes, and in my looking;
God be in my mouth, and in my speaking;
God be in my heart, and in my thinking;
God be at mine end, and at my departing.

It is very devotional, and should be used and heard frequently in the churches throughout the Empire.

### WESTMINSTER ABBEY OUT OF TUNE.

The service concluded, as the *Times* pointedly and properly remarks, *with one verse of the National Anthem.* At St. Paul's the National Anthem is sung, three verses at least being given. At the service to the Red Cross workers in the Abbey, and more especially at the service there last Sunday, the congregation were waiting for the National Anthem to go on and to be completed. There was a general feeling of painful surprise, disappointment, and loss at this formal treatment of our National Anthem by the Abbey authorities. Indeed, their failure once more, to interpret and respond to the feelings of the representatives of all classes of the community, present in the Abbey, was as lamentable as it was unpatriotic.

